# Functional characterization of dopamine and norepinephrine transport across the apical and basal plasma membranes of the human placental syncytiotrophoblast

**DOI:** 10.1038/s41598-022-15790-7

**Published:** 2022-07-08

**Authors:** Hana Horackova, Rona Karahoda, Veronika Vachalova, Helena Turkova, Cilia Abad, Frantisek Staud

**Affiliations:** Department of Pharmacology and Toxicology, Faculty of Pharmacy in Hradec Králové, Charles University, Akademika Heyrovskeho 1203, 500 05 Hradec Králové, Czech Republic

**Keywords:** Transporters in the nervous system, Endocrinology

## Abstract

The human placenta represents a unique non-neuronal site of monoamine transporter expression, with pathophysiological relevance during the prenatal period. Monoamines (serotonin, dopamine, norepinephrine) are crucial neuromodulators for proper placenta functions and fetal development, including cell proliferation, differentiation, and neuronal migration. Accumulating evidence suggests that even a transient disruption of monoamine balance during gestation may lead to permanent changes in the fetal brain structures and functions, projecting into adulthood. Nonetheless, little is known about the transfer of dopamine and norepinephrine across the placental syncytiotrophoblast. Employing the method of isolated membranes from the human term placenta, here we delineate the transport mechanisms involved in dopamine and norepinephrine passage across the apical microvillous (MVM) and basal membranes. We show that the placental uptake of dopamine and norepinephrine across the mother-facing MVM is mediated via the high-affinity and low-capacity serotonin (SERT/SLC6A4) and norepinephrine (NET/SLC6A2) transporters. In the fetus-facing basal membrane, however, the placental uptake of both monoamines is controlled by the organic cation transporter 3 (OCT3/SLC22A3). Our findings thus provide insights into physiological aspects of dopamine and norepinephrine transport across both the maternal and fetal sides of the placenta. As monoamine transporters represent targets for several neuroactive drugs such as antidepressants, our findings are pharmacologically relevant to ensure the safety of drug use during pregnancy.

## Introduction

The human placenta represents a unique non-neuronal site for the metabolism and transport of dopamine, norepinephrine, and serotonin. As messengers on the placenta-brain axis^1^, these monoamine neurotransmitters play essential roles in fetal development and programming. In the early fetal brain, monoamine receptors develop before the inception of synaptogenesis, highlighting their relevance for behavioral development^2^. In addition, they participate in cell proliferation and differentiation processes, neurite outgrowth, modulation of the cell cycle, and neuronal migration^3–7^. Moreover, norepinephrine is a pivotal monoamine for the fetal cardiovascular system, lung function, and glucose mobilization^8,9^ and plays a detrimental role in postnatal adaptation^8^. On the other hand, dopamine affects placental endocrine functions by regulating the production of human placental lactogen and chorionic gonadotropin^10,11^.

Accumulating evidence suggests that even a transient disruption of monoamine balance during gestation may lead to permanent changes in the fetal brain structures and functions, projecting into adulthood^1^. Altered dopamine homeostasis is linked to higher risks of neurological disorders, such as schizophrenia, autism spectrum disorder, bipolar affective, or attention deficit hyperactivity disorder after birth^7,12,13^. In addition, monoamines are vasoactive agents, and inappropriate levels have been related to changes in uterine blood flow, resulting in hypoxia and preterm delivery^14,15^. Thus, the placental transport of monoamines has crucial pathophysiological importance during the prenatal period.

Specific transport proteins of the solute carrier family (SLC) serve as the primary mechanism for the active uptake of monoamines into cells. The three neuronal monoamine transporters—serotonin transporter (SERT/SLC6A4), norepinephrine transporter (NET/SLC6A2), and dopamine transporter (DAT/SLC6A3)—are high-affinity, low-capacity membrane transporters, driven by an inward Na^+^/Cl^−^ gradient and an outward K^+^/H^+^ gradient^16–18^. However, their substrate specificity is not unambiguous, and a significant overlap exists in substrate affinity^19,20^. In addition, a low-affinity but high-capacity organic cation transporter 3 (OCT3/SLC22A3), also known as the extraneuronal monoamine transporter, mediates monoamine uptake in the brain^21,22^. To some extent, monoamines can also pass biological membranes by passive diffusion^23,24^.

The functional unit of the human placenta is the syncytiotrophoblast—an epithelial layer polarized into the apical, maternal-facing microvillous membrane (MVM) and basal, fetal-facing membrane (BM)^25^. Current knowledge of monoamine transport in the placenta is restricted to the MVM, where functional expression of SERT and NET was reported^26,27^. Several studies have failed to detect DAT in placental cells^28–30^, speculating that dopamine must use different transport system(s) to enter the placenta from the maternal circulation. Surprisingly, despite increasing fetal monoamine production towards the term^15^, limited evidence exists on the transport of monoamines across the BM. Our team has recently characterized a serotonin clearance mechanism in the human and rat placenta, mediated by SERT in the MVM and OCT3 in the BM; this synchronized transporter activity in conjunction with the intracellular metabolism by monoamine oxidase A (MAO-A) regulates serotonin levels in the fetoplacental unit^31^. Nonetheless, little is known about the placental handling of dopamine and norepinephrine. The only other report of this kind dates back to 1984; it indicates a non-specific extraction of norepinephrine from the fetal circulation, followed by enzymatic degradation via MAO-A and catechol-o-methyltransferase (COMT)^32^. As OCT3 in neuronal tissues exhibits affinity for dopamine and norepinephrine^33^, we hypothesized that OCT3 might also be responsible for placental uptake of these monoamines from the fetal circulation.

Thus, in the present study, we aimed to characterize dopamine and norepinephrine transport in the human placental syncytiotrophoblast. Experiments were conducted in purified MVM and BM vesicles simultaneously isolated from the human term placenta, providing an ideal system to investigate transport characteristics of both membranes in parallel. Collectively, our findings provide insights into physiological aspects of dopamine and norepinephrine transport across the placenta. As monoamine transporters represent targets for several neuroactive drugs, typically antidepressants, our findings are pharmacologically relevant to ensure the safety of drug use during pregnancy.

## Materials and methods

### Chemicals and reagents

^3^H-norepinephrine, 20 Ci/mmol, ^3^H-serotonin, 80 Ci/mmol, and ^3^H-dopamine, 60 Ci/mmol were purchased from American Radiolabeled Chemicals, Inc. (St. Louis, MO, USA). Dopamine hydrochloride, norepinephrine hydrochloride, serotonin hydrochloride, sodium L-ascorbate, paroxetine hydrochloride, venlafaxine hydrochloride, nisoxetine hydrochloride, GBR 12935 dihydrochloride, decynium-22 and hydrocortisone were purchased from Sigma-Aldrich (St. Louis, MO, USA). Tri Reagent solution was obtained from the Molecular Research Centre (Cincinnati, OH, USA). Bicinchoninic acid assay (BCA assay) reagents were purchased from Thermo Fisher Scientific (Rockford, MA, USA). All other chemicals were of analytical grade.

### Human term placenta sample collection

Term placentas from uncomplicated pregnancies (gestational age at delivery between 39 and 40 weeks) were collected at the University Hospital in Hradec Kralove, Czech Republic. All the experiments conducted in the study conformed to the ethical standards established by the Declaration of Helsinki, and all the women provided signed informed consent. The study protocol was approved by the University Hospital Research Ethics Committee (201006 S15P).

### RNA isolation from human term placenta and reverse transcription

According to the manufacturer's instructions, RNA was isolated from weighed placenta tissue samples using the Tri Reagent solution (Molecular Research Center, Cinncinati, OH, USA). Total RNA concentration and purity were calculated using NanoDrop™ 1000 Spectrophotometer (Thermo Fisher Scientific, Waltham, MA, USA). 1 µg of total RNA was reversely transcribed to cDNA in a total volume of 20 µl using the iScript Advanced cDNA Synthesis Kit (Bio-Rad, Hercules, CA, USA) and Bio-Rad T100™ Thermal Cycler (Hercules, CA, USA), according to the manufacturer's protocol.

### Absolute quantification of *DAT*, *NET*, *SERT*, and *OCT3* transcripts in human term placenta

The quantification of monoamine transporter expression in human term placentas was investigated using duplex droplet digital PCR (ddPCR) analysis, as described previously^31^. Briefly, the duplex reaction mixture consisted of 10 µL ddPCR™ Supermix for probes (Bio-Rad, Hercules, CA, USA), 1 µL of the predesigned probe assay (target gene—FAM, reference gene—HEX), and 1 µL of cDNA (25 ng/µL), in a total volume 20 µL. Expression levels of the following target genes were quantified: *NET/SLC6A2* (Hs00426573_m1), *DAT/SLC6A3* (Hs00997374_m1), *SERT/SLC6A4* (Hs00984349_m1), and *OCT3/SLC22A3* (Hs01009571_m1)—all TaqMan assays were obtained from Thermo Fisher Scientific (Rockford, MA, USA). On the other hand, β-2 microglobulin (*B2M*) was used as the reference gene, obtained from Bio-Rad (Hercules, CA, USA; dHsaCPE5053101). Droplets were created using QX200 Droplet Generator and amplified to the endpoint using the T100™ Thermal Cycler, following the thermal conditions recommended by the manufacturer. Droplets were counted in QX200™ Droplet Reader, and the concentration of the target gene was calculated using the QuantaSoft™ Software. Only samples with more than 13 000 generated droplets were used for final data analysis. Expression levels are reported in the number of transcripts/ng of transcribed RNA. The whole system for ddPCR reaction and analysis was obtained from Bio-Rad (Hercules, CA, USA).

### Preparation of microvillous and basal membrane vesicles from human term placenta

Syncytiotrophoblast plasma membranes were simultaneously prepared from fresh healthy term placentas as described previously^31^. Briefly, maternal decidua and chorionic plate were removed. Next, placental villous tissue (80–100 g) was chopped into small pieces and washed with 0.9% NaCl to remove blood. The purification method involved different steps: homogenization, differential centrifugation, precipitation of non-microvillous membranes with Mg^2+^, and a sucrose gradient step. Microvillous membranes (MVM) and basal membranes (BM) were resuspended in intravesicular buffer (290 mM sucrose, 5 mM Hepes, 5 mM Tris; pH 7.4) and vesiculated by 15 passages through a 25-gauge needle. Protein concentration in the placental homogenate, MVM, and BM vesicles, was analyzed using the BCA assay.

The purity and enrichment of MVM were determined enzymatically by assaying the activity of alkaline phosphatase, according to a well-established protocol^34^. β-adrenergic receptor expressed in the basal membrane was assayed as a basal membrane marker by measuring [^3^H]dihydroalprenolol, levo-[propyl-1,2,3–^3^H] hydrochloride binding^35^. Purity and enrichment results are listed in Supplementary Table S1.

### Monoamine uptake by microvillous and basal membrane vesicles

^3^H-dopamine, ^3^H-norepinephrine, and ^3^H-serotonin uptake by syncytiotrophoblast vesicles (MVM or BM) was performed using the MultiScreen™ Vacuum Manifold 96-well system. Briefly, 3 µL of extravesicular buffer (5 mM Tris, 5 mM HEPES, 145 mM NaCl; pH 7.4) were added into 3 µL of MVM or BM vesicles (10 mg/ml) at room temperature. Uptake was initiated by adding 3 µL of ^3^H-dopamine, 20 µCi/ml (112 nM) or ^3^H-norepinephrine, 20 µCi/ml (332 nM) or ^3^H-serotonin, 24 µCi/ml (100 nM). Uptake was stopped after 1 min or pre-defined time points (time-dependency studies), by adding 200 µL of ice-cold stop solution (130 mM NaCl, 10 mM Na_2_HPO_4_, 4.2 mM KCl, 1.2 mM MgSO_4_, 0.75 mM CaCl_2_; pH 7.4), consequently, transferred into 1 800 µL stop solution and filtered through a 0.45 µm mixed cellulose ester filter (Merck Millipore, Burlington, MA, USA; cat. no. MAHAS4510). Finally, filters were washed ten times with 300 µL of stop solution and cut using the MultiScreen Multiple Punch system (Merck Millipore, Burlington, MA, USA). Protein-free controls were analyzed in parallel to determine tracer binding to the filter, and this background value was subtracted from the total vesicle count.

For concentration-dependent uptake and interaction studies, ^3^H-dopamine, ^3^H-norepinephrine, and ^3^H-serotonin were used as a tracer. The total substrate concentration was supplemented with increasing levels of unlabelled dopamine hydrochloride, norepinephrine hydrochloride, or serotonin hydrochloride. To assess ^3^H-dopamine or ^3^H-norepinephrine binding and/or diffusion in MVM and BM vesicles, uptake experiments were performed at 4 °C at 0 and 1 min incubation times, respectively. On the other hand, to evaluate the effect of intracellular K^+^ on ^3^H-dopamine or ^3^H-norepinephrine uptake, vesicles were preloaded with 140 mM KCl overnight at 4 °C before the uptake experiments.

The effect of specific transport inhibitors on ^3^H-dopamine and ^3^H-norepinephrine uptake was assessed by preincubating MVM and BM vesicles with the selected inhibitors for 10 min at room temperature. Subsequently, ^3^H-dopamine and ^3^H-norepinephrine uptake was assayed in the presence of the inhibitor. The following inhibitors were used: paroxetine 100 µM (SERT, OCT3 inhibitor)^36^, venlafaxine 100 µM (SERT, NET inhibitor)^37^, nisoxetine 20 nM (NET inhibitor)^37^, GBR 12935 50 nM (DAT inhibitor)^38^, hydrocortisone 100 µM (OCT3 inhibitor)^33^, and decynium-22 10 µM (OCT3 inhibitor)^39^. Inhibitors' concentrations were chosen based on previously published data^33,36,40,41^.

### Radioisotope analysis

Filter-associated concentrations of ^3^H-norepinephrine, 20 Ci/mmol, ^3^H-serotonin, 80 Ci/mmol, and ^3^H-dopamine, 60 Ci/mmol were measured by liquid scintillation counting using a Tri-Carb 2910 TR instrument. Non-specific tracer binding to the filter was determined by measuring protein-free controls.

### Statistical analysis

The effect of incubation temperature and intracellular K^+^ on ^3^H-dopamine and ^3^H-norepinephrine uptake by MVM and BM was assessed by the Mann–Whitney test. Kruskal–Wallis test was used to evaluate the effect of inhibitors on ^3^H-dopamine and ^3^H-norepinephrine uptake by MVM and BM. IC_50_ (half-maximal inhibitory concentration) values for monoamine interactions were obtained by fitting concentration-inhibition curves through nonlinear regression analysis. All statistical analyses were implemented in GraphPad prism 9.3.1 software (GraphPad Software, Inc., San Diego, CA, USA). Asterisks in the figures indicate significance levels: * (*p* ≤ 0.05), ** (*p* ≤ 0.01), and *** (*p* ≤ 0.001).

## Results

### Quantification of monoamine transporter transcripts in the human term placenta

ddPCR analysis was employed to determine and compare the transcript levels of monoamine transporters in the human term placenta (Table 1). *NET* expression was predominant among the four monoamine transporters tested, albeit revealing a high inter-individual variability. *SERT* and *OCT3* showed comparable expression levels, whereas *DAT* expression in whole placental tissue was negligible, with transcript levels below 0.2 transcripts/ng RNA.

**Table 1:** Absolute quantification of monoamine transporter gene transcripts in the human term placenta (gestational age at delivery: 39–40).

Gene	Aliases	Transcripts/ng RNA
*SLC6A2*	NET	24.20 (14.28–60.26)
*SLC6A3*	DAT	0.071 (0.030–0.177)
*SLC6A4*	SERT	16.00 (10.50–27.50)
*SLC22A3*	OCT3	5.00 (3.50–7.50)

Target genes were amplified to the endpoint in a duplex ddPCR system, yielding high-precision quantification.

The final output is reported as target transcripts/ng RNA.

The results are shown as median with interquartile range, n ≥ 12.

### Dopamine and norepinephrine uptake by microvillous and basal membrane vesicles isolated from human placental syncytiotrophoblast

**Figure 1:**
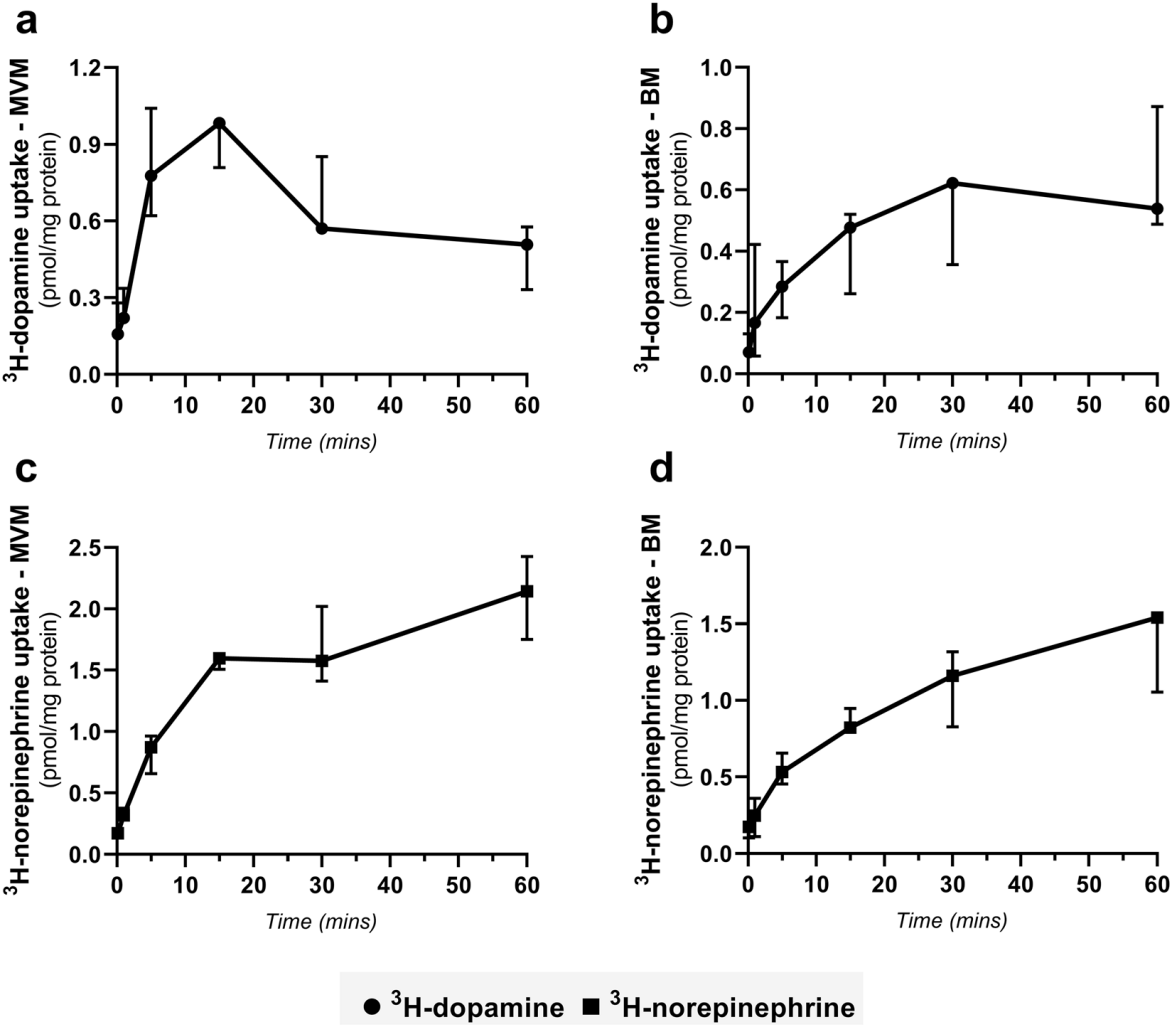
Time-course of ^3^H-dopamine and ^3^H-norepinephrine uptake into MVM and BM vesicles isolated from human term placenta. Vesicle fractions were incubated at room temperature with 112 nM ^3^H-dopamine (**a**, **b**) and 332 nM ^3^H-norepinephrine (**c**, **d**), pH 7.4. The vesicular uptake is presented as pmol/mg protein, and data are shown as median with interquartile range, n = 3.

To characterize dopamine and norepinephrine transport across placental membranes, we conducted uptake studies using ex vivo separately isolated human placenta membrane vesicles (MVM—maternal facing and BM—fetal facing). The time course of ^3^H-dopamine and ^3^H-norepinephrine uptake was determined at pH 7.4 over a period of 20 s to 60 min. As shown in Fig. 1, both membranes exhibited time-dependent ^3^H-dopamine and ^3^H-norepinephrine accumulation. Contrary to ^3^H-norepinephrine, whose uptake increases with time (Fig. 1c), ^3^H-dopamine accumulation by MVM vesicles exhibited the “overshoot phenomenon” (Fig. 1a), previously reported for Na^+^-dependent transporters^29^. At the peak of the overshoot (15 min), the intravesicular ^3^H-dopamine concentration was 1.97 times higher than at 60 min. On the other hand, uptake of ^3^H-dopamine and ^3^H-norepinephrine by BM vesicles was fast and increased with time, reaching equilibrium in 60 min (Fig. 1b, d). Based on these results, a one-minute uptake was selected for subsequent experiments.

**Figure 2:**
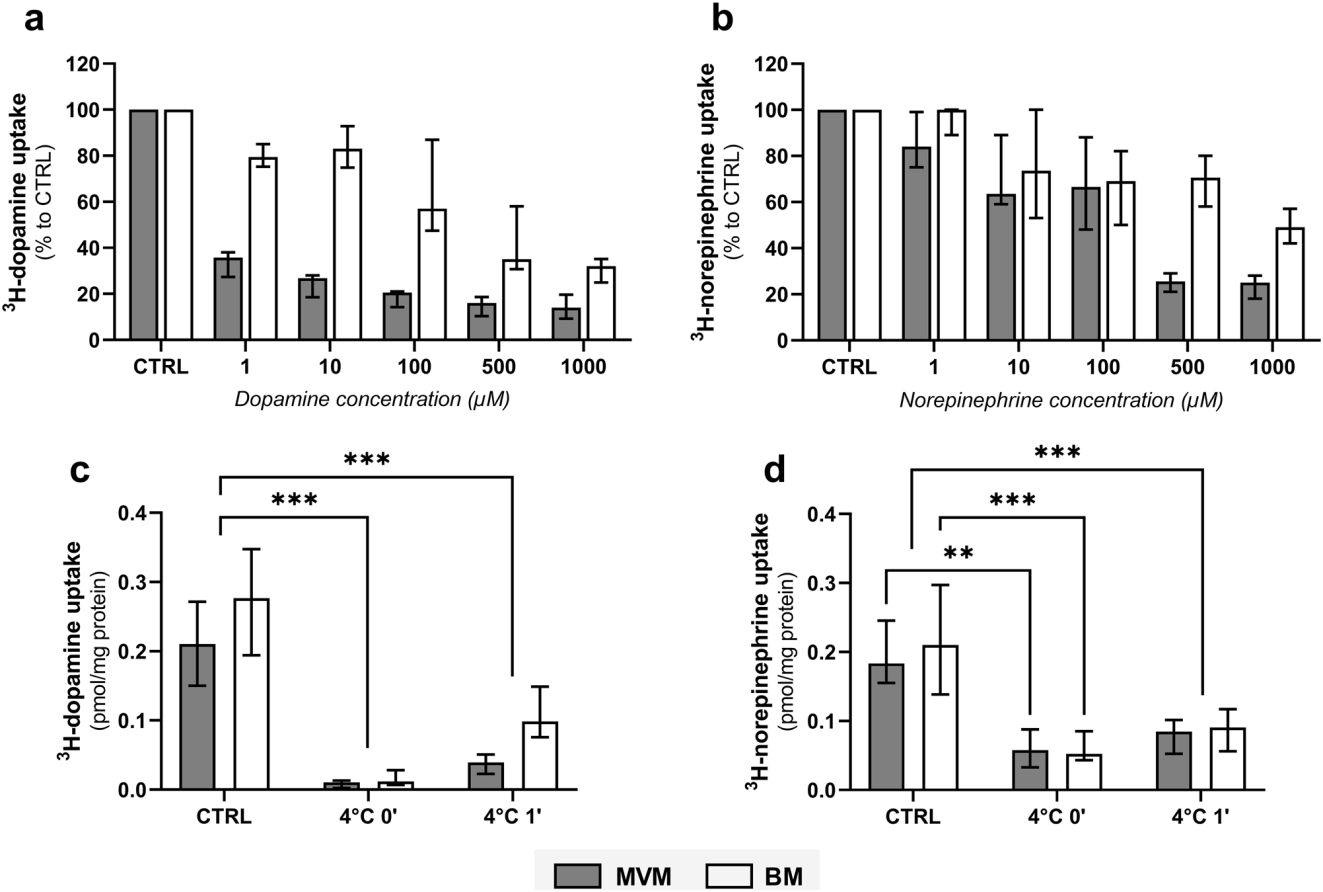
Characterization of ^3^H-dopamine and ^3^H-norepinephrine uptake kinetics into MVM and BM vesicles isolated from human term placenta. Initial uptake rates (CTRL) were determined in vesicles incubated with 112 nM ^3^H-dopamine (**a**, **c**) and 332 nM ^3^H-norepinephrine (**b**, **d**) at room temperature for 1 min. Concentration-dependent analysis of ^3^H-dopamine (**a**) and ^3^H-norepinephrine (**b**) uptakes were evaluated at a range of 1–1000 µM unlabelled substrate concentration. Subsequently, the contribution of non-specific radioisotope binding and/or passive diffusion was determined by analyzing uptake rates of ^3^H-dopamine (**c**) and ^3^H-norepinephrine (**d**) transport at 4 °C (for 0 and 1 min). Results are presented as percent uptake relative to CTRL or as pmol/mg protein. Data are shown as median with interquartile range, n = 4. Statistical analyses were performed using two-way ANOVA: **(*p* ≤ 0.01), ***(*p* ≤ 0.001).

Subsequently, we evaluated the effect of increasing substrate concentration on the intravesicular accumulation of ^3^H-dopamine and ^3^H-norepinephrine, indicating a transporter-mediated uptake mechanism. However, at a concentration range of 1 to 1000 µM, we observed different saturation kinetics between both membranes (Fig. 2a, b). When comparing transporter saturation between MVM and BM, the uptake of ^3^H-dopamine and ^3^H-norepinephrine was more profoundly affected in MVM. These findings indicate that different monoamine transport systems of differing capacities are present on maternal vs fetal side of the placenta. Nonetheless, even at the highest substrate concentration used, we were not able to completely block ^3^H-dopamine and ^3^H-norepinephrine uptake by MVM, and especially BM (Fig. 2a, b). Thus, to evaluate the possibility of non-specific radioisotope binding to the membrane vesicles and/or involvement of passive diffusion, uptake of ^3^H-dopamine and ^3^H-norepinephrine was carried out at 4 °C for 0 and 1 min. Figure 2c shows that ^3^H-dopamine uptake by both membranes at time 0 is negligible. In contrast, compared to control experiments conducted at room temperature, one-minute uptakes at 4 °C recorded 17.9% and 36.3% ^3^H-dopamine accumulation in MVM and BM, respectively (Fig. 2c). This feature suggests potential radioisotope binding and/or a passive diffusion component in the uptake, and this effect is higher in BM vesicles. On the other hand, the fraction of ^3^H-norepinephrine uptake into membrane vesicles at 4 °C was similar in both membranes, regardless of the time (Fig. 2d). This indicates a comparable factor of unspecific ^3^H-norepinephrine binding to the vesicles and/or passive diffusion in both MVM and BM. These findings can explain the unsaturated fraction of ^3^H-dopamine and ^3^H-norepinephrine transport across placental membranes observed in Fig. 2a, b.

### Effect of intracellular K^+^ on dopamine and norepinephrine uptake by placental microvillous and basal membrane vesicles

**Figure 3:**
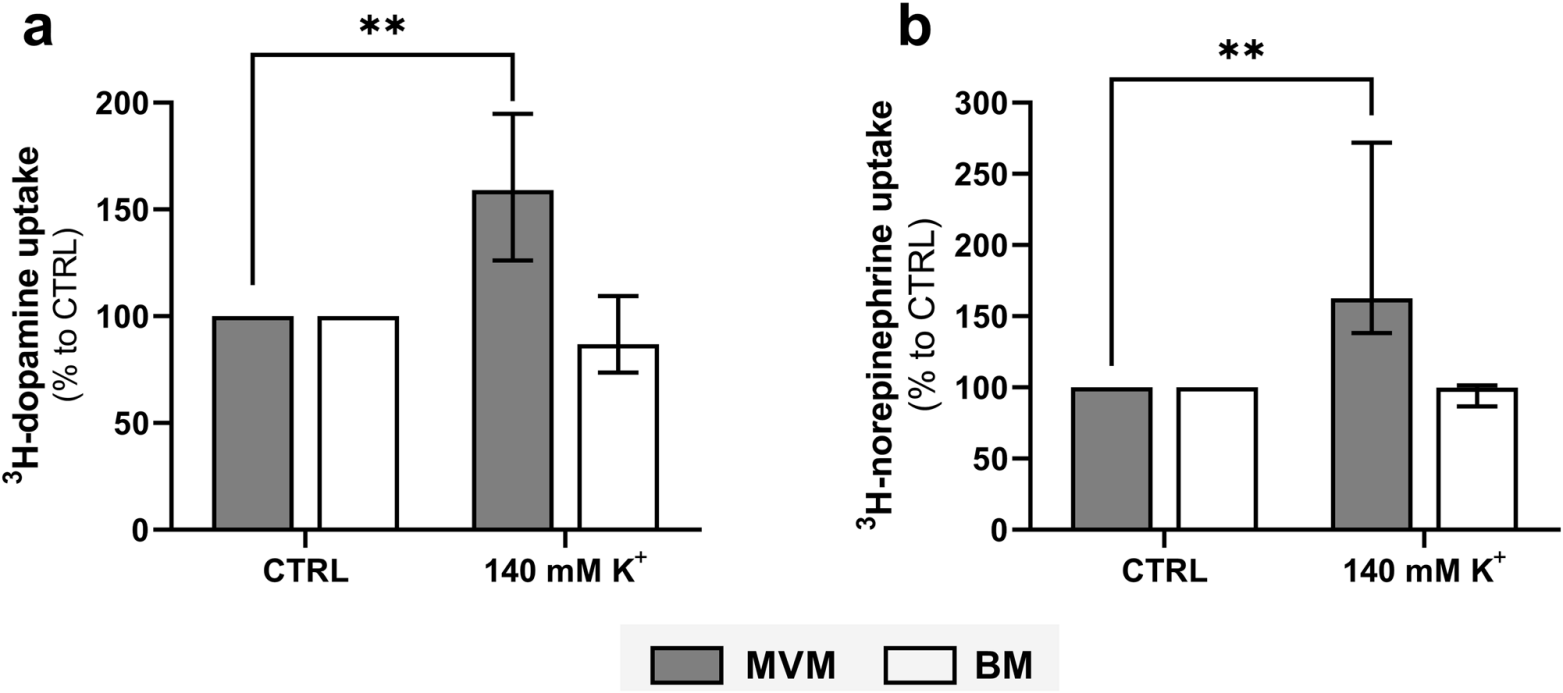
The effect of intracellular K^+^ on monoamine uptake systems in MVM and BM vesicles isolated from human term placenta. Uptake of ^3^H-dopamine (**a**) and ^3^H-norepinephrine (**b**) was determined in membrane vesicles preloaded overnight with 140 mM K^+^. Results are presented as percent uptake relative to the unloaded vesicles (CTRL). Data are expressed as median with interquartile range, n = 4. Statistical analyses were performed using Mann–Whitney test: **(*p* ≤ 0.01).

To distinguish between the neuronal (DAT, NET, SERT) and extraneuronal (OCT3) monoamine transporters in MVM and BM, we investigated the effect of intracellular K^+^ on dopamine and norepinephrine uptake. This is because neuronal monoamine transporters are driven by an extracellular Na^+^/Cl^−^ and an intracellular K^+^/H^+^ concentration gradient. Furthermore, substrate uptake by DAT and NET is reportedly voltage-dependent due to the transient nature of intracellular K^+^ binding^42^. We observed that overnight loading of MVM vesicles with 140 mM K^+^ significantly increased the uptake of ^3^H-dopamine and ^3^H-norepinephrine (Fig. 3a). On the other hand, radioisotope accumulation in BM was unaffected by the intracellular K^+^ concentration (Fig. 3b). These results confirm the presence of different catecholamine transport mechanisms in MVM and BM vesicles, with the MVM displaying an electrogenic transport system for dopamine and norepinephrine.

### Identification of dopamine and norepinephrine transport systems in MVM and BM using specific transport inhibitors

**Figure 4:**
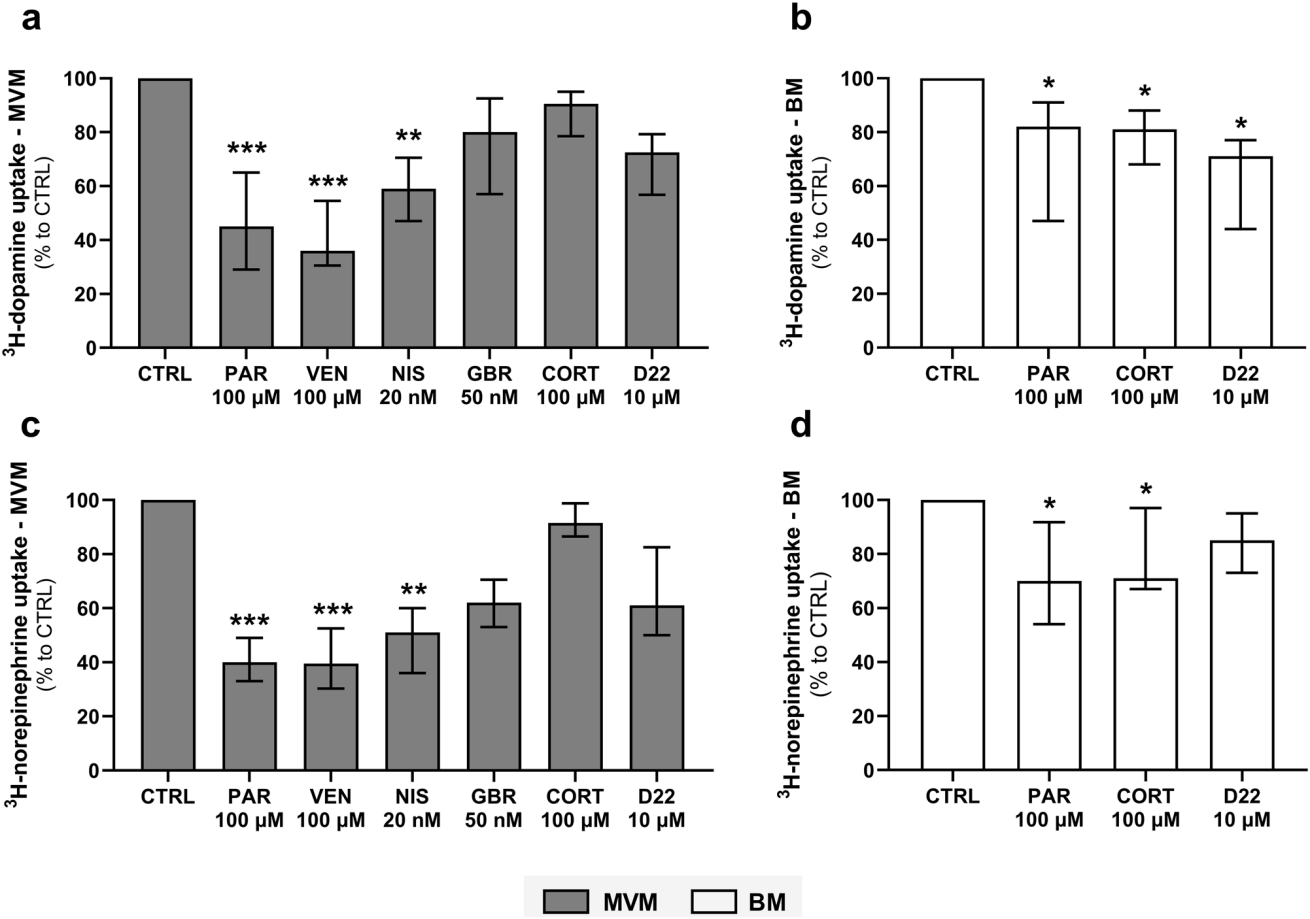
Differential inhibitor sensitivity of ^3^H-dopamine and ^3^H-norepinephrine transport systems in human placental membranes. One-minute uptake of ^3^H-dopamine (**a**, **b**) and ^3^H -norepinephrine (**c**, **d**) was evaluated in the presence of 100 µM paroxetine (PAR), 100 µM venlafaxine (VEN), 20 nM nisoxetine (NIS), 50 nM GBR 12935 (GBR), 100 µM cortisol (CORT), and 10 µM decynium-22 (D22). Results are presented as percent uptake relative to the uptake in the absence of inhibitors (CTRL). Data are shown as median with interquartile range, n ≥ 4. Statistical analysis was evaluated using Kruskal–Wallis test: *(*p* ≤ 0.05), **(*p* ≤ 0.01), ***(*p* ≤ 0.001).

A selection of monoamine transporter inhibitors was assayed to further characterize the transporters responsible for dopamine and norepinephrine uptake by MVM and BM vesicles. Since there is no involvement of neuronal transporters (NET, DAT, SERT) in the BM, only inhibitors related to the extraneuronal transport system (OCT3) were tested in this membrane. We found that paroxetine, venlafaxine, and nisoxetine significantly inhibit ^3^H-dopamine (Fig. 4a) and ^3^H-norepinephrine (Fig. 4c) uptake by the MVM vesicles. These data highlight SERT and NET as the predominant transporters involved in dopamine and norepinephrine membrane transport by MVM. On the other hand, paroxetine and cortisol displayed weak albeit significant inhibitory action on ^3^H-dopamine (Fig. 4b) and ^3^H-norepinephrine (Fig. 4d) uptake by the BM vesicles. Additionally, BM uptake of ^3^H-dopamine was affected by decynium-22. Collectively, these results suggest that OCT3 mediates the transport of ^3^H-dopamine and ^3^H-norepinephrine across the BM.

### Characterization of the dynamic interactions of monoamines with placental transport systems

**Figure 5:**
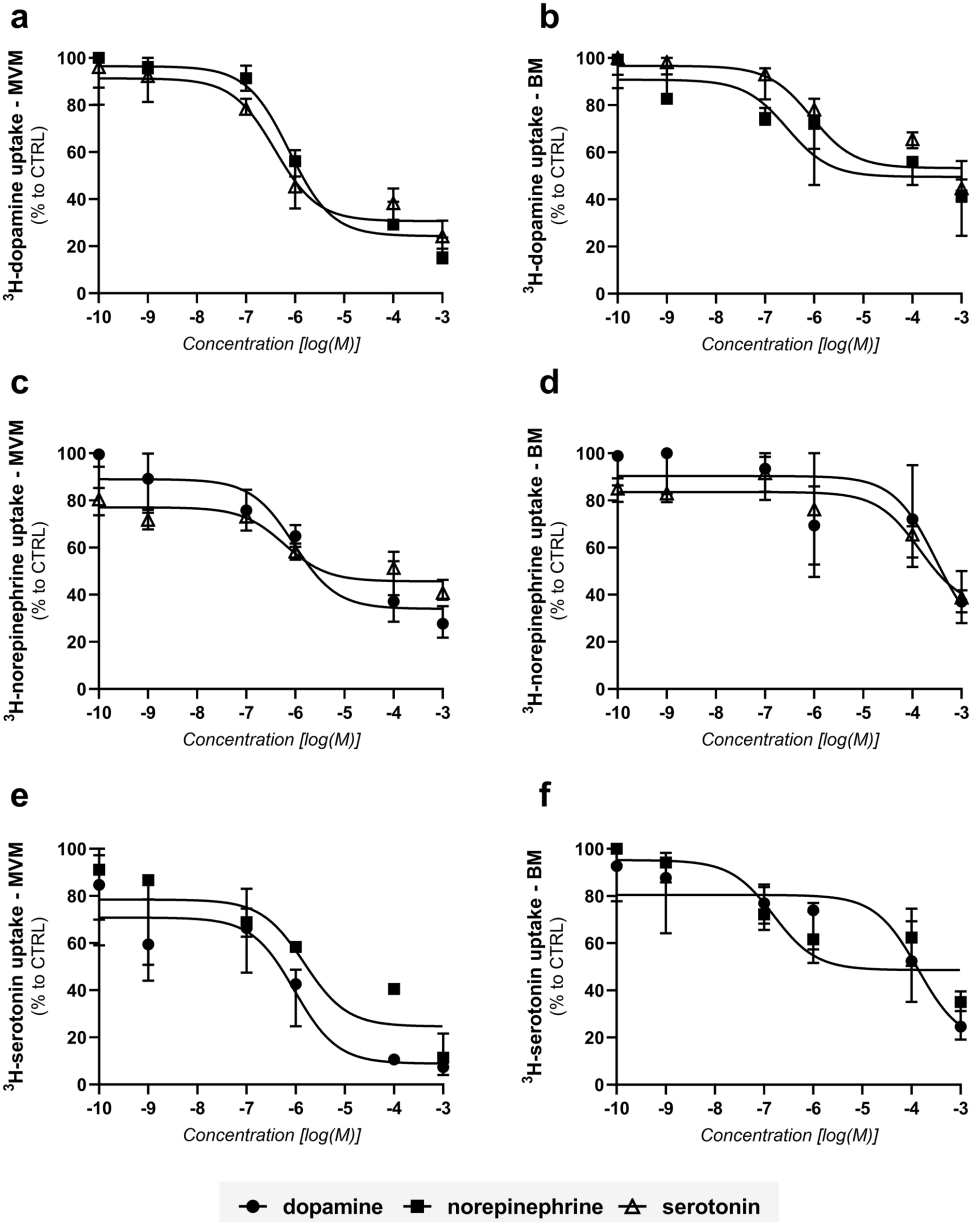
Effect of unlabelled dopamine, norepinephrine, and serotonin on monoamine transport systems in MVM and BM. Initial uptake rates (CTRL) were determined in vesicles incubated with 112 nM ^3^H-dopamine (**a**, **b**), 332 nM ^3^H-norepinephrine (**c**, **d**), and 100 nM ^3^H-serotonin (**e**, **f**) at room temperature for 1 min. Concentration-dependent analyses of unlabelled monoamines were evaluated at a range of 0.0001–1000 µM. Curve fitting by nonlinear regression was implemented in GraphPad Prism 8.1. Results are presented as percent uptake relative to CTRL. Data are shown as median with interquartile range, n = 4.

To investigate the interactions of monoamines on placental monoamine transporters, the effect of unlabelled monoamines on ^3^H-dopamine, ^3^H-norepinephrine, and ^3^H-serotonin uptake was investigated (Fig. 5a–f). These experiments suggest that the individual monoamine transport across placental MVM and BM does not occur via a single transporter, and a significant interaction between the three monoamines is observed on both membranes. ^3^H-dopamine (Fig. 5a) and ^3^H-norepinephrine (Fig. 5c) uptake by MVM was most potently inhibited by serotonin, whereas dopamine was the most potent inhibitor of ^3^H-serotonin (Fig. 5e) uptake by this membrane. In the BM vesicles, norepinephrine was the most potent monoamine to affect ^3^H-dopamine (Fig. 5b) and ^3^H-serotonin uptake (Fig. 5f). The half-maximal inhibitory concentrations (IC_50_) values are shown in Table 2.

**Table 2:** IC_50_ values (nM) for inhibition of 112 nM ^3^H-dopamine, 332 nM ^3^H-norepinephrine, and 100 nM ^3^H-serotonin uptake by MVM and BM in the presence of increasing concentrations of unlabelled monoamines.

IC_50_ (nM)	MVM			BM		
	Dopamine	Norepinephrine	Serotonin	Dopamine	Norepinephrine	Serotonin
^3^H-dopamine	–	766	389	–	283	885
^3^H-norepinephrine	939	–	602	3056	–	1422
^3^H-serotonin	948	1491	–	1357	154	–

## Discussion

**Figure 6:**
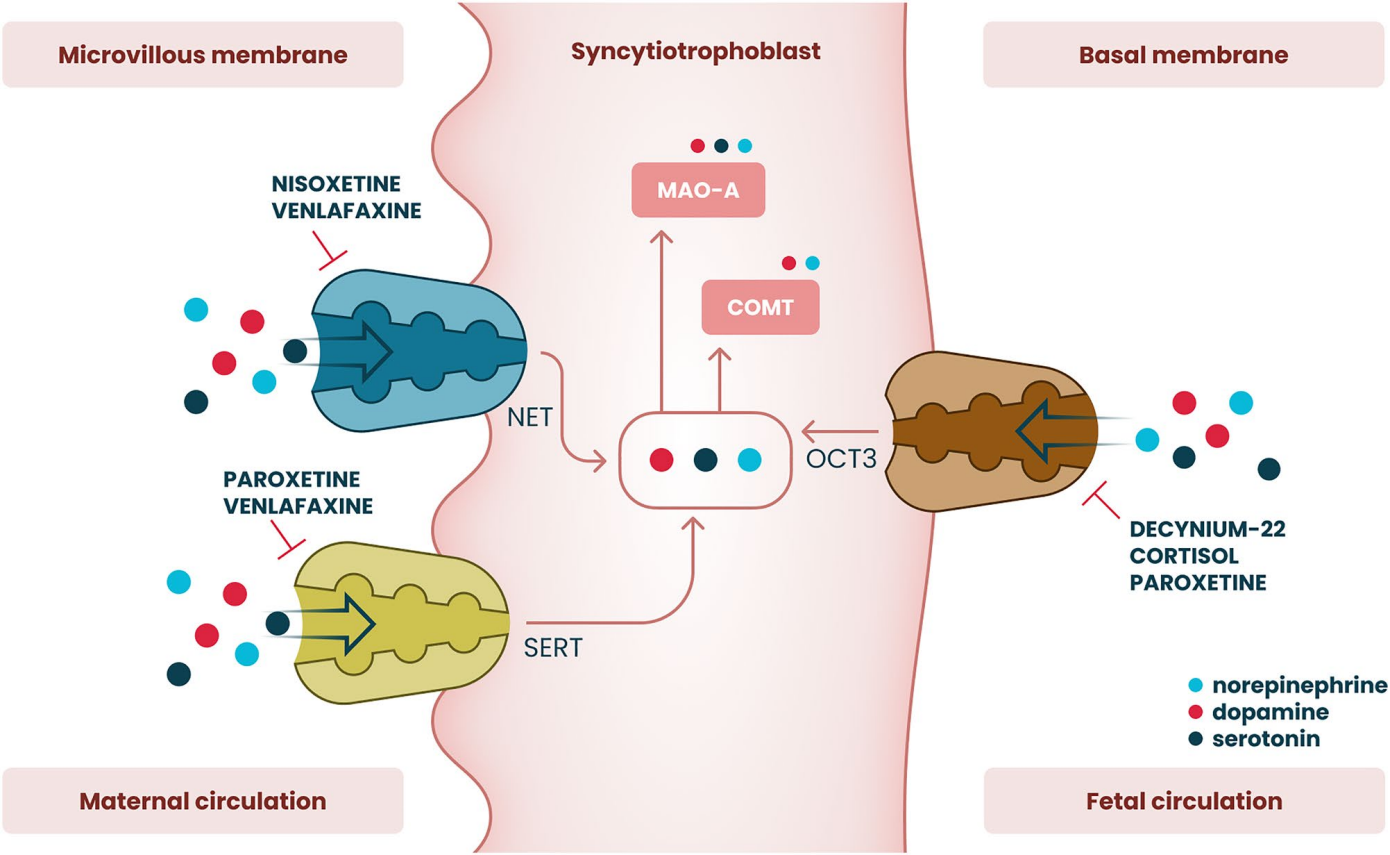
Summary of monoamine transport mechanisms in the apical and basal membranes of the human placental syncytiotrophoblast. Active uptake of dopamine and norepinephrine by the microvillous membrane is mediated via the high-affinity and low-capacity serotonin (SERT/SLC6A4) and norepinephrine (NET/SLC6A2) transporters. On the other hand, the organic cation transporter 3 (OCT3/SLC22A3) contributes to the placental monoamine clearance system by effectively mediating dopamine and norepinephrine uptake from the fetal circulation across the basal membrane.

This study characterizes the transport mechanisms involved in dopamine and norepinephrine transfer across the apical and basal membranes of the human placenta (Fig. 6). Uptake by syncytiotrophoblast represents one of the main mechanisms of monoamine handling in the fetoplacental unit, and intrauterine catecholamine clearance is primarily mediated via transporter-dependent mechanisms^43^. Our findings expand on the pioneering work by Ganapathy et al., who were the first to identify the functional expression of monoamine transporters in the placenta^44^. The importance of SERT and NET for maternal-placental handling of dopamine and norepinephrine has been clearly established. However, despite increasing fetal monoamine production towards term^15^, limited attempts have been made to elucidate dopamine and norepinephrine transport across the fetal-facing basal membrane. Using an established ex vivo model of human placenta membrane vesicles, we demonstrate a novel physiological mechanism of dopamine and norepinephrine transport across the fetal-facing membrane into the placenta by OCT3.

Increasing evidence supports the placenta as a dopaminergic and adrenergic organ^1^. Although polar molecules, dopamine and norepinephrine were reported to cross the placental barrier from the maternal to the fetal circulation^45,46^. Moreover, increased fetal monoamine secretion during pregnancy results in elevated cord blood levels^15^. This intrauterine neuroendocrine milieu requires a finely tuned clearance mechanism to control monoamine levels in the fetoplacental unit. The current state of knowledge has primarily focused on the MVM, where functional expression of neuronal monoamine transporters (SERT and NET) is recognized as critical to intrauterine monoamine homeostasis^26,27^. Our findings show that these transporters share a similar transcriptional profile in the human placenta. In agreement with previous studies^29^, we demonstrate that NET mediates norepinephrine uptake by MVM in a time-, concentration- and Na^+^-dependent manner. Nonetheless, inhibition of norepinephrine transport in the presence of paroxetine (a selective SERT inhibitor) indicates the potential involvement of SERT in total norepinephrine uptake by MVM, although with significantly lower affinity.

In contrast, DAT expression in the human placenta is negligible and, based on the functional studies, does not contribute to monoamine uptake. This was previously suggested by Ramamoorthy et al., who attributed dopamine transport across placental MVM to NET^30^. Interestingly, we observed that dopamine uptake by MVM vesicles reveals an overshoot phenomenon over time, which largely mirrors that of serotonin, previously reported by our team^31^. On the contrary, norepinephrine uptake does not exhibit such a phenomenon, thus, suggesting SERT as the key transporter responsible for dopamine uptake by MVM. However, the sensitivity of the uptake to selective SERT (paroxetine) and NET (nisoxetine) inhibitors suggests an overlapping substrate specificity whereby both SERT and NET contribute to placental uptake of dopamine across the maternal-facing membrane. Indeed, accumulating evidence reveals that the SLC6A members share structural and mechanistic properties^47,48^. Consequently, dopamine has already been described as an alternative substrate of SERT and NET in neuronal tissue, albeit with a mechanistically distinct mode of substrate translocation^49,50^.

Importantly, our findings reveal a concentration-dependent placental uptake of dopamine and norepinephrine across the fetal-facing BM. Isotope displacement kinetics revealed different profiles between MVM and BM, indicating the involvement of a high-capacity transporter in BM. Moreover, intracellular potassium concentrations did not affect dopamine and norepinephrine uptake by BM vesicles, thus excluding the involvement of neuronal monoamine transporters in the fetus-facing membrane. We have recently shown that the low-affinity but high-capacity OCT3 mediates placental serotonin uptake from the fetal circulation^31^. Since OCT3 recognizes dopamine and norepinephrine as substrates^33^, we speculated that this transporter might also mediate the uptake of these two monoamines. Using several OCT3 inhibitors^33,39^, we confirm that this transporter is involved in the placental uptake of fetal dopamine and norepinephrine. Nonetheless, we identified a non-saturable fraction in the transport kinetics across BM. Although not widely acknowledged in the literature, several studies indicate that dopamine and norepinephrine may cross biological barriers also by passive diffusion^23,24^. Such a mechanism was also previously speculated in the placenta^32^, particularly in the BM, where water permeability through a lipid diffusive mechanism is significantly higher than in MVM^51^.

Fetal-to-maternal transfer of norepinephrine was first described by Sodha et al. using a human placenta perfusion system. The authors demonstrated that up to 50% of extracted fetal norepinephrine is metabolized within the trophoblast via COMT and MAO-A and released on the maternal side in the form of inactive metabolites^32^. Later, this was also reported in the ovine model, highlighting the role of the placenta in fetal norepinephrine clearance^52^. Similarly, we have shown that via a synchronized activity of transporters (SERT and OCT3) and metabolizing enzyme (MAO-A), the placenta contributes significantly to serotonin clearance from the fetal circulation^31^. Thus, we propose that the human placenta plays a neutralizing role in preventing elevated monoamine levels in the fetoplacental unit under physiological conditions.

Monoamine transporter promiscuity in the placenta was previously suggested in the MVM^44^; here, we describe a similar behavior also in the BM. It is thus logical to assume potential competition of monoamines for their transporters on both the maternal and fetal sides of the placenta. However, we report that the calculated IC_50_ values are notably higher than the physiological plasma concentrations of these monoamines in healthy pregnant women^53–55^, suggesting that mutual interactions and competition for uptake systems do not occur under physiological conditions. Nonetheless, this phenomenon may be detrimental in placental pathologies such as preeclampsia, associated with altered monoamine transporter expression^27^ and elevated plasma monoamine concentrations^54–56^. Moreover, inhibition of monoamine transporters by illicit drugs (e.g., cocaine) and pharmaceuticals (e.g., antidepressants) used during pregnancy, or endogenous molecules (e.g., cortisol), could compromise monoamine levels in the fetoplacental unit^29,36^, contributing to the adverse effect on fetal development and programming.

The strength of this study is using simultaneously isolated MVM and BM from the same placenta, thus minimizing the influence of clinical and genetic heterogeneity when comparing the transporter characteristics in each membrane. Nonetheless, while isolated human placental membranes provide an ideal model to investigate syncytial transport aspects (including ion composition, transport kinetics, and effect of inhibitors), this is done in the absence of intracellular constituents and regulatory factors^57^. Thus, an intact cell system is required to identify the contribution of specific transporter(s) on monoamine transplacental flux in vivo. Moreover, cellular metabolism should be considered when estimating the total clearance capacity.

In conclusion, the placenta provides an efficient clearance system for dopamine and norepinephrine from both maternal and fetal circulations. Although not investigated in this study, we speculate that this transport function plays a multifaceted role in placental physiology in conjunction with intracellular metabolism. Ultimately, placental monoamine transport and metabolism may constitute essential mechanisms implicated in fetal programming of adulthood disorders.

## Supplementary Information

Supplementary Table S1.

## Data Availability

All data generated or analyzed during this study are included in this published article (and its Supplementary Information files).
