# Supplementary material for: Functional characterization of dopamine and norepinephrine transport across the apical and basal plasma membranes of the human placental syncytiotrophoblast
Source: Sci Rep. 2022 Jul 8;12:11603. doi: 10.1038/s41598-022-15790-7 (PMC9270497; doi:10.1038/s41598-022-15790-7)
Supplement: Supplementary file 1 — Supplementary Table S1. [file 41598_2022_15790_MOESM1_ESM.pdf]

SUPPLEMENTARY INFORMATION

FUNCTIONAL CHARACTERIZATION OF DOPAMINE AND NOREPINEPHRINE TRANSPORT ACROSS THE APICAL AND BASAL PLASMA MEMBRANES OF THE HUMAN PLACENTAL SYNCYTIOTROPHOBLAST

Hana Horackova, Rona Karahoda, Veronika Vachalova, Helena Turkova, Cilia Abad, Frantisek Staud

Table S1. Enrichment and orientation of *ex vivo* isolated microvillous (MVM) and basal (BM) membranes from healthy human term placenta. Data are shown as means ± SD, n≥4. Alkaline phosphatase and dihydroalprenolol binding were calculated against total placental homogenates.

Parameter	MVM	BM
Orientation (% right side out)	95.02 ± 6.39	87.22 ± 4.89
Alkaline phosphatase (fold enrichment)	19.31 ± 7.90	4.58 ± 0.30
Dihydroalprenolol binding (fold enrichment)	8.21 ± 4.51	31.08 ± 9.84
